# Sample size estimation using a latent variable model for mixed outcome co‐primary, multiple primary and composite endpoints

**DOI:** 10.1002/sim.9356

**Published:** 2022-02-23

**Authors:** Martina E. McMenamin, Jessica K. Barrett, Anna Berglind, James M. S. Wason

**Affiliations:** ^1^ MRC Biostatistics Unit University of Cambridge Cambridge UK; ^2^ WHO Collaborating Centre for Infectious Disease Epidemiology and Control, School of Public Health The University of Hong Kong Hong Kong Special Administrative Region China; ^3^ Late Respiratory & Immunology, Biometrics, BioPharmaceuticals R& D AstraZeneca Gothenburg Sweden; ^4^ Population Health Sciences Institute Newcastle University Newcastle upon Tyne UK

**Keywords:** latent variable modeling, mixed outcome endpoints, sample size estimation

## Abstract

Mixed outcome endpoints that combine multiple continuous and discrete components are often employed as primary outcome measures in clinical trials. These may be in the form of co‐primary endpoints, which conclude effectiveness overall if an effect occurs in all of the components, or multiple primary endpoints, which require an effect in at least one of the components. Alternatively, they may be combined to form composite endpoints, which reduce the outcomes to a one‐dimensional endpoint. There are many advantages to joint modeling the individual outcomes, however in order to do this in practice we require techniques for sample size estimation. In this article we show how the latent variable model can be used to estimate the joint endpoints and propose hypotheses, power calculations and sample size estimation methods for each. We illustrate the techniques using a numerical example based on a four‐dimensional endpoint and find that the sample size required for the co‐primary endpoint is larger than that required for the individual endpoint with the smallest effect size. Conversely, the sample size required in the multiple primary case is similar to that needed for the outcome with the largest effect size. We show that the empirical power is achieved for each endpoint and that the FWER can be sufficiently controlled using a Bonferroni correction if the correlations between endpoints are less than 0.5. Otherwise, less conservative adjustments may be needed. We further illustrate empirically the efficiency gains that may be achieved in the composite endpoint setting.

## 1 INTRODUCTION

Sample size estimation plays an integral role in the design of a study. The objective is to determine the minimum sample size that is large enough to detect, with a specified power, a clinically meaningful treatment effect. Although it is crucial that investigators have enough patients enrolled to detect this effect, overestimating the sample size also has ethical and practical implications. Namely, in a placebo‐controlled trial, more patients are subjected to a placebo arm than is necessary, therefore withholding access to potentially beneficial drugs from them and delaying access to future patients.^1^, ^2^, ^3^ Furthermore it results in longer, more expensive trials, using resources that could be allocated elsewhere.

One vital aspect of sample size determination is the primary endpoint. Typically this is a single outcome, however in some instances there may be multiple outcomes of interest and so various combinations of these outcomes can be selected as the primary endpoint, depending on the hypothesis of interest. Assuming we have three outcomes of interest $\nu_{1},\nu_{2}$ and $\nu_{3}$, one option is a co‐primary endpoint, which takes the form of the multivariate endpoint $\nu_{1}\cap \nu_{2}\cap \nu_{3}$. This means that an intervention is deemed to be effective overall if it is shown to be effective in each of $\nu_{1},\nu_{2}$, and $\nu_{3}$. Alternatively multiple primary endpoints may be of interest, which take the multivariate form $\nu_{1}\cup \nu_{2}\cup \nu_{3}$, where an intervention is deemed effective if it is shown to be effective in at least one of $\nu_{1},\nu_{2}$, or $\nu_{3}$. Another possibility is a composite endpoint, involving some function that maps the multivariate outcome to a univariate outcome for inference, for example $\nu_{1}+\nu_{2}+\nu_{3}$. In this case the outcomes within the composite may be assigned equal or differing degrees of relevance depending on clinical importance.^4^ Alternatively, the composite endpoint may combine outcomes by labeling patients as ‘responders’ or ‘non‐responders’ based on whether they exceed predefined thresholds in each of the outcomes. For instance, we let a response indicator S=1 if $\nu_{1}\leq \eta_{1},\nu_{2}\leq \eta_{2}$, and $\nu_{3}\leq \eta_{3}$, where η denotes the response cutpoints. Note that the composite case is distinct from the others in that it combines the parameters and hence test statistics for each outcome into one, rather than these remaining separate for each outcome. This will have implications for sample size estimation.

For each of these endpoints, the individual outcomes may be a mix of multiple continuous, ordinal, and binary measures. One possible way to jointly model the outcomes is using a latent variable framework, arising in the graphical modeling literature, in which discrete outcomes are assumed to be latent continuous variables subject to estimable thresholds and modeled using a multivariate normal distribution.^5^, ^6^ By employing this framework we can take account of the correlation between the outcomes, improve the handling of missing data in individual components and potentially increase efficiency. Furthermore, in the case of multiple primary outcomes, it may reduce the severity of multiple testing corrections required by accounting for correlation between endpoints.

A barrier to adopting these techniques is a lack of consensus on sample size determination. A recent and comprehensive overview of the existing literature for sample size calculation in clinical trials with co‐primary and multiple primary endpoints is provided by Sozu et al.^7^ The review found many proposals for power and sample size calculations for multiple continuous outcomes. In the co‐primary case, some of these were based on assuming that the endpoints were bivariate normally distributed,^8^, ^9^ and extended for the case of more than two endpoints.^10^, ^11^ Other work focused on testing procedures^12^, ^13^ and controlling the type I error rate.^14^, ^15^, ^16^, ^17^ Similar ideas were investigated for multiple primary endpoints.^14^, ^17^, ^18^, ^19^

Approaches to sample size estimation for composite endpoints have focused primarily on the case of multiple binary components.^20^, ^21^, ^22^, ^23^, ^24^, ^25^ In the case of binary co‐primary endpoints, five methods of power and sample size calculation based on three association measures have been introduced.^26^ Additionally, sample size calculation for trials using multiple risk ratios and odds ratios for treatment effect estimation is discussed by Hamasaki et al,^27^ and Song^28^ explores co‐primary endpoints in non‐inferiority clinical trials. Consideration has also been given to the case where two co‐primary endpoints are both time‐to‐event measures where effects are required in both endpoints,^29^, ^30^, ^31^ and at least one of the endpoints.^32^, ^33^ Furthermore, composites comprised of time‐to‐event measures are common, in which the composite reflects time‐to‐first‐event variable.^34^ Sample size estimation in this case has been considered by Sugimoto et al.^35^

The mixed outcome setting has received substantially less consideration. One paper considers overall power functions and sample size determinations for multiple co‐primary endpoints that consist of mixed continuous and binary variables.^36^ They assume that response variables follow a multivariate normal distribution, where binary variables are observed in a dichotomized normal distribution, and use Pearson's correlations for association. A modification was suggested by Wu and de Leon^37^ which involved using latent‐level tests and pairwise correlations, and provided increased power. These methods focus on the co‐primary endpoint case, where effects are required in all outcomes. The case of multiple primary or composite endpoints where the components are measured on different scales has not been considered, each of which will require distinct hypotheses. In practice, if a mixed outcome composite is selected as the primary endpoint in a trial then the sample size calculation may be based on an overall binary endpoint or collapsed to form multiple binary endpoints however this will result in a large loss in efficiency.^38^

In this article we build on the existing work for co‐primary continuous and binary endpoints to include any combination of continuous, ordinal, and binary outcomes for co‐primary, multiple primary, and composite endpoints. We propose a framework based on the same latent variable model and show how it may be tailored to each of the three endpoints to facilitate sample size estimation. The article will proceed as follows: in Section 2 we introduce the latent variable model, detailing how it can be used in each context, and specify hypothesis tests for each of the three combinations of mixed outcomes; in Section 3 we propose power calculations and sample size estimation techniques in each case; in Section 4 we illustrate the methods on a four dimensional endpoint consisting of two continuous, one ordinal and one binary outcome using a numerical example based on the MUSE trial;^39^ and in Section 5 we simulate the empirical power for each test and the FWER for the union‐intersection test. We conclude with a discussion and recommendations for practice in Section 6, and introduce user‐friendly software and documentation for implementation in Section 7.

## 2 ENDPOINTS AND HYPOTHESIS TESTING

### 2.1 Latent variable framework

Let $n_{T}$ and $n_{C}$ represent the number of patients in the treatment group and the control group respectively and let *K* be the number of outcomes measured for each patient. Let $Y_{\mathrm{Ti}}={\left(Y_{Ti1},\ldots ,Y_{TiK}\right)}^{T},i=1,\ldots ,n_{T}$ be vector of *K* responses for patient *i* on the treatment arm and $Y_{\mathrm{Ci}}={\left(Y_{Ci1},\ldots ,Y_{CiK}\right)}^{T},i=1,\ldots ,n_{C}$ the vector of *K* responses for patient *i* on the control arm. Without loss of generality, the first $1\leq k\leq k_{m}$ elements of $Y_{\mathrm{Ti}}$ and $Y_{\mathrm{Ci}}$ are observed as continuous variables, the next $k_{m}<k\leq k_{o}$ are observed as ordinal and the remaining $k_{o}<k\leq K$ are observed as binary. For instance, for a three dimensional endpoint with one continuous, one ordinal and one binary measure, $k_{m}=1,k_{o}=2$, and K=3. We use the biserial model of association by Tate,^40^ which is based on latent continuous measures manifesting as discrete variables. Formally, we say that $Y_{\mathrm{Ti}}$ and $Y_{\mathrm{Ci}}$ have latent variables $Y_{\mathrm{Ti}}^{*}$ and $Y_{\mathrm{Ci}}^{*}$ respectively, where $Y_{\mathrm{Ti}}^{*}∼N_{K}(\mu_{T},\sum_{T})$ and $Y_{\mathrm{Ci}}^{*}∼N_{K}(\mu_{C},\sum_{C})$, where $\mu_{T}=(\mu_{1T},\ldots \mu_{KT})$, $\mu_{kT}=\mu_{kT0}+\mu_{kT1}x_{kT1}+\cdots +\mu_{kTp}x_{kTp}$ and $x_{kT1}...x_{kTp}$ denotes the *p* covariates included in the model for outcome *k*. Likewise $\mu_{C}=(\mu_{1C},\ldots \mu_{KC})$ are the corresponding quantities for the control arm. Then for $k\neq k^{\prime }:1\leq k<k^{\prime }\leq k_{m}$ let $Var(Y_{Tik})=\sigma_{Tk}^{2},Var(Y_{Cik})=\sigma_{Ck}^{2}$ and $Corr(Y_{Tik},Y_{Tik^{\prime }})=\rho_{Tkk^{\prime }},Corr(Y_{Cik},Y_{Cik^{\prime }})=\rho_{Ckk^{\prime }}$, where $\rho_{Tkk^{\prime }}$ and $\rho_{Ckk^{\prime }}$ are the association measures between the endpoints. For $k_{m}<k\leq K$, $Var(Y_{Tik}^{*})=Var(Y_{Cik}^{*})=1$ and $Corr(Y_{Tik}^{*},Y_{Tik^{\prime }}^{*})=\rho_{Tkk^{\prime }}^{*},Corr(Y_{Cik}^{*},Y_{Cik^{\prime }}^{*})=\rho_{Ckk^{\prime }}^{*}$. The latent variables can be related to the observed variables by:

- $1\leq k\leq k_{m}:Y_{Tik}=Y_{Tik}^{*}$ and $Y_{Cik}=Y_{Cik}^{*}$
- $k_{m}<k\leq k_{o}:$
$Y_{Tik}=\left\{\begin{array}{cc} 0\; & \text{if}\;\tau_{k0}\leq Y_{Tik}^{*}<\tau_{k1},\\ 1\; & \text{if}\;\tau_{k1}\leq Y_{Tik}^{*}<\tau_{k2},\\ \vdots \; & \;\vdots \\ w_{k}\; & \text{if}\;\tau_{kw_{k}}\leq Y_{Tik}^{*}<\tau_{k(w_{k}+1)} \end{array}\right.Y_{Cik}=\left\{\begin{array}{cc} 0\; & \text{if}\;\tau_{k0}\leq Y_{Cik}^{*}<\tau_{k1},\\ 1\; & \text{if}\;\tau_{k1}\leq Y_{Cik}^{*}<\tau_{k2},\\ \vdots \; & \;\vdots \\ w_{k}\; & \text{if}\;\tau_{kw_{k}}\leq Y_{Cik}^{*}<\tau_{k(w_{k}+1)} \end{array}\right.$
- $k_{o}<k\leq K:Y_{Tik}=\left\{\begin{array}{cc} 0\; & \text{if}\;\tau_{k0}\leq Y_{Tik}^{*}<\tau_{k1},\\ 1\; & \text{if}\;\tau_{k1}\leq Y_{Tik}^{*}<\tau_{k2} \end{array}\right.\;Y_{Cik}=\left\{\begin{array}{cc} 0\; & \text{if}\;\tau_{k0}\leq Y_{Cik}^{*}<\tau_{k1},\\ 1\; & \text{if}\;\tau_{k1}\leq Y_{Cik}^{*}<\tau_{k2} \end{array}\right.$

We set $\tau_{k0}=-\infty ,\tau_{k(w_{k}+1)}=\infty$ and the intercepts $\mu_{kT0}$ and $\mu_{kC0}$ equal to zero for $k_{m}<k\leq k_{o}$ in order to estimate the cut‐points. Additionally, $\tau_{k0}=-\infty ,\tau_{k1}=0,\tau_{k2}=\infty$ for $k_{o}<k\leq K$ so that the intercepts can be estimated for the outcomes observed as binary. The mixed outcomes are then combined as follows.

### 2.2 Co‐primary endpoint

In this case, a treatment must be shown to be effective as measured by each of the outcomes in order to be deemed effective overall. We generalize previous work for mixed continuous and binary outcomes to include ordinal outcomes, as shown below.^36^, ^37^ In many clinical trials the hypothesis of interest is based on superiority, namely that the proposed treatment will perform better than the control treatment. The null hypothesis is that the difference in treatment effects for the treatment arm and control arm is less than or equal to zero. This is straightforward to formalize in the case of one endpoint but less so when there are multiple co‐primary endpoints, particularly when they are measured on different scales. The hypothesis of interest is as shown in (1)

(1) $$\begin{array}{ll} & H_{0}:\;\exists \;k\;s.t.\;\pi_{Tk}-\pi_{Ck}\leq 0\\ & H_{1}:\;\pi_{Tk}-\pi_{Ck}>0\;\forall k, \end{array}$$

where $\pi_{Tk}$ and $\pi_{Ck}$ is the effect of the intervention in the treatment and control arm respectively. For $k_{o}<k\leq K$ we can specify $\pi_{Tik}=P(Y_{Tik}=0)=P(Y_{Tik}^{*}<0)$ and $\pi_{Cik}=P(Y_{Cik}=0)=P(Y_{Cik}^{*}<0)$ for the treatment and control group.

We can generalize this assumption to account for the ordinal endpoints based on the fact that for $k_{m}<k\leq k_{o}$ $\pi_{Tik}=P(Y_{Tik}=w_{k})=P(\tau_{kw_{k}}<Y_{Tik}^{*}<\tau_{k(w_{k}+1)})$. The definition of treatment effect for ordinal outcomes may be modified to include multiple ordinal levels by selecting the appropriate τ thresholds. For instance, $\pi_{Tik}=P(Y_{Tik}=0)+P(Y_{Tik}=1)+P(Y_{Tik}=2)=P(-\infty <Y_{Tik}^{*}<\tau_{k3})$. As the latent means are estimable by maximum likelihood, $\mu_{Ti1}^{*}=\Phi^{-1}(\pi_{Ti1}),\ldots ,\mu_{TiK}^{*}=\Phi^{-1}(\pi_{TiK})$ in the treatment group and $\mu_{Ci1}^{*}=\Phi^{-1}(\pi_{Ci1}),\ldots ,\mu_{CiK}^{*}=\Phi^{-1}(\pi_{CiK})$ in the control group.

We can proceed by specifying that the hypothesis in (1) holds if and only if the hypothesis

(2) $$\begin{array}{ll} & H_{0}^{*}:\;\exists \;k\;s.t.\;\delta_{k}^{*}\leq 0\\ & H_{1}^{*}:\;\delta_{k}^{*}>0\;\forall k, \end{array}$$

holds, where $\delta_{k}^{*}=\mu_{Tk}^{*}-\mu_{Ck}^{*}$, $\mu_{Tk}^{*}=1/n_{T}\sum_{i=1}^{n_{T}}\mu_{Tik}^{*}$ and $\mu_{Ck}^{*}=1/n_{C}\sum_{i=1}^{n_{C}}\mu_{Cik}^{*}$. The maximum likelihood estimates ${\hat{\mu }}_{Tk}^{*}$ and ${\hat{\mu }}_{Ck}^{*}$ can be used for a test of $H_{0}^{*}$ and the variance of this test statistic can be obtained using the inverse of the Fisher information matrix.

### 2.3 Multiple primary endpoint

Multiple primary endpoints conclude a treatment is effective if it is shown to work in at least one of the outcomes. We would expect the sample size required to be reduced compared with the co‐primary endpoint case which would require power to detect treatments in all outcomes. We can allow for sample size estimation for multiple primary endpoints as follows.

The hypothesis of interest, accounting for the fact that a significant effect in only one outcome is required, is shown below.

(3) $$\begin{array}{ll} & H_{0}:\;\pi_{Tk}-\pi_{Ck}\leq 0\;\forall k\\ & H_{1}:\;\exists \;k\;s.t.\;\pi_{Tk}-\pi_{Ck}>0. \end{array}$$

As before, $\pi_{Tk}$ and $\pi_{Ck}$ can be determined for $k_{m}<k\leq k_{o}$ using the relevant τ thresholds.

(4) $$\begin{array}{ll} & H_{0}^{*}:\;\delta_{k}^{*}\leq 0\;\forall k\\ & H_{1}^{*}:\;\exists \;k\;s.t.\;\delta_{k}^{*}>0. \end{array}$$

The difference in latent means $\delta_{k}^{*}=\mu_{Tk}^{*}-\mu_{Ck}^{*}$ and their variance are estimated using the maximum likelihood estimates and Fisher information matrix, as before.

### 2.4 Composite endpoint

A review conducted by Wason et al^41^ showed that composite responder endpoints are widely used and identified many clinical areas in which they are common, such as oncology, rheumatology, cardiovascular, and circulation. The latent variable framework may be used to model the underlying structure of these mixed outcome composite endpoints to greatly improve efficiency.^38^ The joint distribution of the continuous, ordinal, and binary outcomes is modeled using the latent variable structure as before. However, in this case the endpoint of interest is a composite responder endpoint and so the required quantity is some function of the probability of response in the treatment group $p_{T}$ and in the control group $p_{C}$.

For instance, an overall responder index $S_{i}$ can be formed for patient *i*, where $S_{i}=1$ if $Y_{i1}\leq \eta_{1},\ldots ,Y_{iK}^{*}\leq \eta_{K}$ and 0 otherwise, where the quantities $\left(\eta_{1},\ldots ,\eta_{K}\right)$ are predefined responder thresholds. Generalizations where response only requires a certain number of the components to meet the thresholds are possible, but involve more complex sums. Note that this definition of response is distinct from that commonly found in composites formed from survival endpoints or binary composites typical in cardiovascular studies. We can specify $p_{iT}$ and $p_{iC}$, the probability of response for patient *i* in the treatment arm and control arm respectively, as shown in (5),

(5) $$\begin{array}{ll} p_{iT}=P(S_{i}=1|T_{i}=1) & =\int_{-\infty }^{\eta_{1}}\ldots \int_{-\infty }^{\eta_{K}}f_{Y_{1},\ldots ,Y_{K}}(y_{i1},\ldots ,y_{iK}|T_{i}=1,\theta )dy_{K}\ldots dy_{1}\\ p_{iC}=P(S_{i}=1|T_{i}=0) & =\int_{-\infty }^{\eta_{1}}\ldots \int_{-\infty }^{\eta_{K}}f_{Y_{1},\ldots ,Y_{K}}(y_{i1},\ldots ,y_{iK}|T_{i}=0,\theta )dy_{K}\ldots dy_{1}, \end{array}$$

where θ is the vector of model parameters and we assume that $p_{T}∼N(\delta_{T},\sigma_{\delta_{T}}^{2})$ and $p_{C}∼N(\delta_{C},\sigma_{\delta_{C}}^{2})$. As in the case of co‐primary and multiple primary endpoints, the assumptions allow us to estimate latent means $\left(\mu_{k_{m+1}}^{*},\ldots ,\mu_{K}^{*}\right)$ for the observed discrete components using the model parameters.

In the mixed outcome composite endpoint setting, note that although we are exploiting the latent multivariate Gaussian structure for efficiency gains we are ultimately still interested in a one dimensional endpoint, such as the difference in response probabilities between the treatment and control arms of the trial. This is distinct from the co‐primary and multiple primary endpoints cases, where the overall hypothesis test must be based on some union or intersection of the hypotheses for the individual outcomes. For the composite endpoint we can formulate the hypothesis as shown in (6),

(6) $$\begin{array}{ll} & H_{0}:\;p_{T}-p_{C}\leq 0\\ & H_{1}:\;p_{T}-p_{C}>0, \end{array}$$

where $p_{T}$ and $p_{C}$ are as in (5). For sample size estimation, we require the distribution of $\delta =p_{T}-p_{C}$ under $H_{1}$, which we can assume to be $\delta ∼N(\delta_{T}-\delta_{C},\sigma_{\delta }^{2})$. The hypothesis can therefore be stated as

(7) $$\begin{array}{ll} & H_{0}:\;\delta^{*}\leq 0\\ & H_{1}:\;\delta^{*}>0, \end{array}$$

where $\delta^{*}=\delta_{T}^{*}-\delta_{C}^{*}$, $\delta_{T}^{*}=\Phi_{K}(\eta_{1},\ldots ,\eta_{K};\mu_{T}^{*},\sum_{T})$, $\delta_{C}^{*}=\Phi_{K}(\eta_{1},\ldots ,\eta_{K};\mu_{C}^{*},\sum_{C})$ and $\Phi_{K}(.;\mu ,\sum )$ is the K‐dimensional multivariate normal distribution function, with mean vector μ and covariance matrix ∑. Estimates of the quantities can be obtained using the maximum likelihood estimates for the model parameters, as in the co‐primary and multiple primary endpoint settings, so that ${\hat{\delta }}_{T}^{*}=\Phi_{K}(\eta_{1},\ldots ,\eta_{K};{\hat{\mu }}_{T}^{*},{\hat{\sum }}_{T})$ and ${\hat{\delta }}_{C}^{*}=\Phi_{K}(\eta_{1},\ldots ,\eta_{K};{\hat{\mu }}_{C}^{*},{\hat{\sum }}_{C})$, where ${\mu_{T}}^{*}$ is the K‐dimensional vector of mean values in the treatment arm and ${\mu_{C}}^{*}$ is the corresponding vector for the control arm. Using a Taylor series expansion, we can obtain the quantity $\sigma_{\delta }^{2}$ using the fact that $var({\hat{\delta }}^{*})\approx {{(}^{\prime \prime }\delta )}^{T}Cov(\hat{\theta }){(}^{\prime \prime }\delta )$. Then, $\hat{var}({\hat{\delta }}^{*})={{(}^{\prime \prime }\delta_{T})}^{T}\hat{Cov}(\hat{\theta }){(}^{\prime \prime }\delta_{T})$, where ${}^{\prime \prime }\delta$ is the vector of partial derivatives of $\delta^{*}$ with respect to each of the parameter estimates. We can obtain $\hat{\theta }$ and covariance matrix $\hat{Cov}(\hat{\theta })$ by fitting the model to pilot trial data.

## 3 SAMPLE SIZE ESTIMATION

### 3.1 Co‐primary endpoints

To construct the power function, we define the required quantities as follows. Let ${\bar{Y}}_{Tk}-{\bar{Y}}_{Ck}$ and ${\hat{\mu }}_{Tk}^{*}-{\hat{\mu }}_{Ck}^{*}$ denote the difference in sample means for the continuous and discrete outcomes respectively. We assume $\delta_{k}=\mu_{Tk}-\mu_{Ck}$, $\delta_{k}^{*}=\mu_{Tk}^{*}-\mu_{Ck}^{*}$, $\kappa =n_{C}/n_{T}$ and let $z_{\alpha }$ denote the (1−α)100th standard normal percentile, where α is the prespecified significance level. We define the *z* score as $Z_{k}=\frac{{\bar{Y}}_{Tk}-{\bar{Y}}_{Ck}}{\sigma_{k}\sqrt{\frac{1+\kappa }{\kappa n_{T}}}}$ and $Z_{k}^{*}=\frac{{\hat{\mu }}_{Tk}^{*}-{\hat{\mu }}_{Ck}^{*}}{\sqrt{\frac{1+\kappa }{\kappa n_{T}}}}$ for the observed continuous and latent continuous measures respectively. The test statistic can then be defined as shown below.

(8) $$Z_{k}^{†}=\left\{\begin{array}{cc} Z_{k}-\frac{\delta_{k}}{\sigma_{k}}\sqrt{\frac{\kappa n_{T}}{1+\kappa }}=\frac{{\bar{Y}}_{Tk}-{\bar{Y}}_{Ck}-\delta_{k}}{\sigma_{k}\sqrt{\frac{1+\kappa }{\kappa n_{T}}}}, & k=1,\ldots ,k_{m}\\ Z_{k}^{*}-\delta_{k}^{*}\sqrt{\frac{\kappa n_{T}}{1+\kappa }}=\frac{{\hat{\mu }}_{Tk}^{*}-{\hat{\mu }}_{Ck}^{*}-\delta_{k}^{*}}{\sqrt{\frac{1+\kappa }{\kappa n_{T}}}}, & k=k_{m+1},\ldots ,K \end{array}\right.\;,$$

(9) $$z_{k}^{†}=\left\{\begin{array}{cc} z_{\alpha }-\frac{\delta_{k}}{\sigma_{k}}\sqrt{\frac{\kappa n_{T}}{1+\kappa }}, & k=1,\ldots ,k_{m}\\ z_{\alpha }-\delta_{k}^{*}\sqrt{\frac{\kappa n_{T}}{1+\kappa }}, & k=k_{m+1},\ldots ,K \end{array}\right.\;.$$

A useful property of $Z^{†}={\left(Z_{1}^{†},\ldots Z_{K}^{†}\right)}^{T}$ is that it is asymptotically multivariate normal under regularity conditions.^11^ The power function for the joint co‐primary endpoints is as shown in (10) and hence can be approximated by (11).

(10) $$1-\beta =P\left({⋂}_{k=1}^{k_{m}}\{Z_{k}>z_{\alpha }\}{⋂}_{k_{m+1}}^{K}\{Z_{k}^{*}>z_{\alpha }\}\;|\delta \right)≃P\left({⋂}_{k=1}^{K}\{Z_{k}^{†}>z_{k}^{†}\}\;|\delta \right),$$

for $\delta ={\left(\delta_{1},\ldots ,\delta_{k_{m}},\ldots ,\delta_{k_{o}},\ldots ,\delta_{K}\right)}^{T}\neq 0$.

(11) $$1-\beta ≃P\left({⋂}_{k=1}^{K}\{Z_{k}^{†}>z_{k}^{†}\}\;|\delta \right)=\Phi_{K}\left(-z_{1}^{†},\ldots ,-z_{K}^{†};\Gamma \right).$$

Assuming $n_{T}=n_{C}=n$ it is possible to rearrange (11) to obtain a sample size formula in terms of *n* as shown below.^7^

(12) $$n=\frac{{\left(C_{K}+z_{\alpha }\right)}^{2}}{\delta_{K}^{2}},$$

where the sample size depends on the number of outcomes and $C_{K}$ is the solution of

(13) $$1-\beta =\int_{-\infty }^{\gamma_{1}C_{K}+z_{\alpha }(\gamma_{1}-1)}\ldots \int_{-\infty }^{\gamma_{K-1}C_{K}+z_{\alpha }(\gamma_{K-1}-1)}\int_{-\infty }^{C_{K}}f\left(z_{1},\ldots ,z_{K}^{*};0,\Gamma \right)dz_{K}^{*}\ldots dz_{1}.$$

Alternatively, we can input different values for *n* in (11) to achieve the required power.

### 3.2 Multiple primary endpoints

Using the $Z_{k}^{†}$ and $z_{k}^{†}$ defined for co‐primary endpoints and assuming $n_{T}=n_{C}=n$, we can define the overall power as in (14).

(14) $$1-\beta =P\left({⋃}_{k=1}^{k_{m}}\{Z_{k}>z_{\alpha }\}{⋃}_{k_{m+1}}^{K}\{Z_{k}^{*}>z_{\alpha }\}\;|\delta \right)≃P\left({⋃}_{k=1}^{K}\{Z_{k}^{†}>z_{k}^{†}\}\;|\delta \right).$$

In order to obtain an appropriate power function we rely on the inclusion‐exclusion principle as follows.

$$\begin{array}{llll} P\left({⋃}_{k=1}^{K}\{Z_{k}^{†}>z_{k}^{†}\}\;|\delta \right) & =\overset{K}{\underset{k=1}{\sum }}P\left(\{Z_{k}^{†}>z_{k}^{†}\}\;|\delta \right)-\underset{k<l}{\sum }P\left(\{Z_{k}^{†}>z_{k}^{†}\}\cap \{Z_{l}^{†}>z_{l}^{†}\}\;|\delta \right)\; & & \\ & \;+\underset{k<l<m}{\sum }P\left(\{Z_{k}^{†}>z_{k}^{†}\}\cap \{Z_{l}^{†}>z_{l}^{†}\}\cap \{Z_{m}^{†}>z_{m}^{†}\}\;|\delta \right)\; & & \\ & \;+\cdots +{\left(-1\right)}^{K-1}\underset{k<\cdots <K}{\sum }P\left({⋂}_{k=1}^{K}\{Z_{k}^{†}>z_{k}^{†}\}\;|\delta \right).\; & & \end{array}$$

A closed form expression for the overall power is shown in (15).

(15) $$P\left({⋃}_{k=1}^{K}\{Z_{k}^{†}>z_{k}^{†}\}\;|\delta \right)=\overset{K}{\underset{i=1}{\sum }}\left({\left(-1\right)}^{i-1}\underset{I\subseteq \{1,\ldots ,K\}}{\sum }P\left({⋂}_{k\in I}\{Z_{k}^{†}>z_{k}^{†}\}\;|\delta \right)\right).$$

We then input different values for *n* to achieve the required power. Note when using the union‐intersection test for multiple primary endpoints that a correction must be applied to control the family‐wise error rate (FWER). Approaches used for multiple primary continuous endpoints, such as Bonferroni and Holm corrections, may also be implemented in this setting.

### 3.3 Composite endpoints

As the endpoint of interest is specified in terms of the overall one dimensional composite endpoint, we can use the formula assumed when employing the standard test of proportions technique. As $\sigma_{\delta }=\sqrt{\frac{\sigma_{\delta_{T}}^{2}}{n_{T}}+\frac{\sigma_{\delta_{C}}^{2}}{n_{C}}}$, we can assume that $\sigma_{T}=\sigma_{C}=\sigma$ and $n_{T}=n_{C}=n$, so that $\delta ∼N(\delta_{T}-\delta_{C},2\sigma^{2}/n)$. The power is deduced in the standard way, as demonstrated below.

(16) $$\begin{array}{ll} 1-\beta & =P\left({\bar{p}}_{T}-{\bar{p}}_{C}>z_{\alpha }\sqrt{2\sigma^{2}/n}|H_{1}\right)\\ & =P\left(Z>\frac{z_{\alpha }\sqrt{2\sigma^{2}/n}-\delta^{*}}{\sqrt{2\sigma^{2}/n}}|H_{1}\right)\\ & =\Phi \left(\frac{\delta^{*}}{\sqrt{2\sigma^{2}/n}}-z_{\alpha }\right). \end{array}$$

Note that $\sigma_{\delta }^{2}=\frac{2\sigma^{2}}{n}$, however to obtain a formula in terms of the required sample size we will need to separate *n* from the variance estimate. By fitting the model to pilot trial data we can obtain an estimate for $\sigma^{2}$, as the value of *n* will be known in this instance and *n* can be obtained using (17).

(17) $$n=\frac{2{\hat{\sigma }}^{2}{\left(z_{1-\beta }+z_{\alpha }\right)}^{2}}{{\hat{\delta }}^{*2}}.$$

This is similar to the sample size equation used for the binary method, however σ is not derived in the standard way and $\delta^{*}$ is obtained using latent means as opposed to provided directly.

## 4 NUMERICAL APPLICATION

### 4.1 MUSE trial

We illustrate the technique for sample size determination using the MUSE trial.^39^ The trial was a phase IIb, randomized, double‐blind, placebo‐controlled study investigating the efficacy and safety of anifrolumab in adults with moderate to severe systemic lupus erythematosus (SLE). Patients (*n*=305) were randomized (1:1:1) to receive anifrolumab (300 or 1000 mg) or placebo, in addition to standard therapy every 4 weeks for 48 weeks. The primary endpoint in the study was the percentage of patients achieving an SLE Responder Index (SRI) response at week 24, with sustained reduction of oral corticosteroids (<10 mg/day and less than or equal to the dose at week 1 from week 12 through 24). SRI is comprised of a continuous Physician's Global Assessment (PGA) measure, a continuous SLE Disease Activity Index (SLEDAI) measure and an ordinal British Isles Lupus Assessment Group (BILAG) measure.^42^ The study had a target sample size of 100 patients per group based on providing 88% power at the two‐sided 0.10 alpha level, to detect at least 20% absolute improvement in SRI(4) response rate at week 24 for anifrolumab relative to placebo. The investigators assumed a 40% placebo response rate.

### 4.2 Model

In this case $\left(Y_{1},Y_{2},Y_{3},Y_{4}\right)$ are SLEDAI, PGA, BILAG and the corticosteroid tapering indicator respectively and $\left(Y_{1},Y_{2},Y_{3}^{*},Y_{4}^{*}\right)∼N_{4}\left(\mu^{*},\sum \right)$ where,

(18) $$\begin{array}{ll} \mu^{*}={\left(\mu_{1},\mu_{2},\mu_{3}^{*},\mu_{4}^{*}\right)}^{T}\;\sum =\left(\begin{array}{cccc} \sigma_{1}^{2} & \rho_{12}\sigma_{1}\sigma_{2} & \rho_{13}\sigma_{1} & \rho_{14}\sigma_{1}\\ \rho_{12}\sigma_{1}\sigma_{2} & \sigma_{2}^{2} & \rho_{23}\sigma_{2} & \rho_{24}\sigma_{2}\\ \rho_{13}\sigma_{1} & \rho_{23}\sigma_{2} & 1 & \rho_{34}\\ \rho_{14}\sigma_{1} & \rho_{24}\sigma_{2} & \rho_{34} & 1 \end{array}\right), & \end{array}$$

and the ordinal and binary components may be related to their latent variables as shown in (19). The thresholds $\left(\tau_{31},\tau_{32},\tau_{33},\tau_{34}\right)$ are estimated from the data.

(19) $$Y_{i3}=\left\{\begin{array}{cc} 0\; & \text{if}\;-\infty <Y_{i3}^{*}<\tau_{31},\\ 1\; & \text{if}\;\tau_{31}\leq Y_{i3}^{*}<\tau_{32},\\ 2\; & \text{if}\;\tau_{32}\leq Y_{i3}^{*}<\tau_{33},\\ 3\; & \text{if}\;\tau_{33}\leq Y_{i3}^{*}<\tau_{34},\\ 4\; & \text{if}\;\tau_{34}\leq Y_{i3}^{*}<\infty , \end{array}\right.\;Y_{i4}=\left\{\begin{array}{cc} 0\; & \text{if}\;-\infty <Y_{i4}^{*}<0,\\ 1\; & \text{if}\;0\leq Y_{i4}^{*}<\infty \; \end{array}\right.$$

We can use the MUSE trial to design future studies where we assume that the endpoints of interest are co‐primary, multiple primary and composite endpoints. The overall power functions for each are shown below.

$$\begin{array}{ll} & Power_{co}=\Phi_{4}\left(-z_{1}^{†},-z_{2}^{†},-z_{3}^{†},-z_{4}^{†};\sum \right)\\ & Power_{mult}=\overset{4}{\underset{i=1}{\sum }}\left({\left(-1\right)}^{i-1}\underset{I\subseteq \{1,2,3,4\}}{\sum }\Phi_{k\in I}\left(-z_{k}^{†};\sum \right)\right)\\ & Power_{comp}=\Phi \left(-z\right), \end{array}$$

where $z_{k}^{†}=z_{\alpha }-\frac{\delta_{k}}{\sqrt{2\sigma_{k}^{2}/n}}$ for k={1,2} and $z_{k}^{†}=z_{\alpha }-\frac{\delta_{k}^{*}}{\sqrt{2/n}}$ for k={3,4}. In the composite setting $z=\frac{\delta^{*}}{\sqrt{2\sigma^{2}/n}}-z_{\alpha }$ where σ is estimated using the delta method. For the $Power_{mult}$ calculation we apply the Bonferroni correction, such that each outcome is assessed at the $\frac{\alpha }{4}$ level.

### 4.3 Computation

We have conducted the computations in R version 4.0.2. We define functions to evaluate the power for each of the endpoints using a combination of the pnorm and pmvnorm functions. Sample size is obtained by inserting values for n until the desired power is achieved. Details of our source code and a web app for implementation is included in the Software section. Code to obtain the results shown in this article can be obtained at https://github.com/martinamcm/mcmenamin_2021_multsamp. Considerations and instructions for fitting the latent variable model are discussed in detail McMenamin et al. (2021).^38^

### 4.4 Results

**FIGURE 1:**
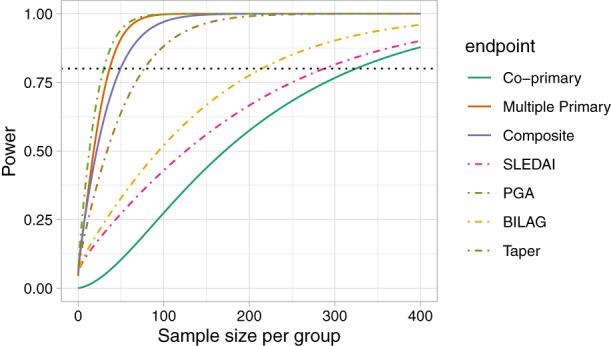
Power function for individual SLEDAI (continuous), PGA (continuous), BILAG (ordinal), and Taper (binary) outcomes and the power functions with when they are treated as co‐primary, multiple primary, and composite endpoints using data from the MUSE trial

The power is largest for the multiple primary endpoint, where 80% is achieved for n=37 patients in each arm. The power for the composite endpoint is similar to that of PGA, the component with the highest effect size. As we would expect the power is considerably lower for co‐primary endpoints, which would require n=325 for 80% power (Figure 1).

**FIGURE 2:**
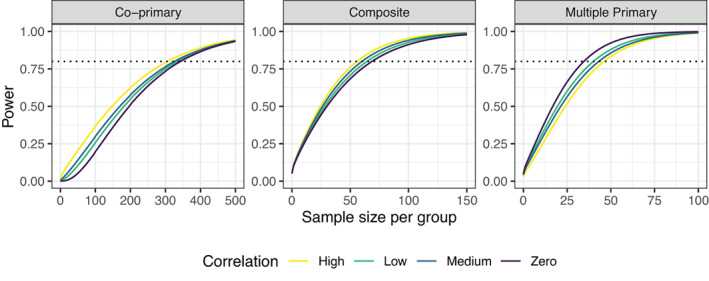
Overall power 1−β to detect the treatment effects assumed from the MUSE trial for the systemic lupus erythematosus co‐primary, multiple primary, and composite endpoints for different sample sizes per group $n=n_{C}=n_{T}$ and differing correlations between outcomes, where Low = 0.3, Medium = 0.5, and High = 0.8

Table 1 shows the sample sizes required in each group, for the co‐primary and multiple primary endpoints to obtain an overall power of at least 80% to detect a difference of 0.88 in SLEDAI, 0.38 in PGA, 0.24 in BILAG and 0.40 in the taper outcome based on the values observed in the trial. We allow for uncertainty in the variance of the continuous measures by setting $\sigma_{1}^{2}=18,19,20$ and $\sigma_{2}^{2}=0.35,0.45,0.55,0.65$. The sample sizes required for each individual endpoint are also shown, based on achieving a power of at least 80%. Allowing for uncertainty in the variance of the SLEDAI outcome varies the required sample size for the co‐primary endpoint but not the multiple primary endpoint. The opposite is true when the assumed variance of the PGA outcome is changed, namely affecting the sample size required for the multiple primary endpoint but not the co‐primary. This is intuitive given that the treatment effect observed in the SLEDAI outcome is smallest and is largest for the PGA outcome. For the co‐primary and composite endpoints the power is largest when the correlation between the endpoints is high whereas for multiple primary endpoints the power is largest for zero correlation between endpoints (Figure 2).

**TABLE 1:** Sample sizes $n=n_{C}=n_{T}$ for the co‐primary and multiple primary endpoints for overall power 1−β≈0.80, α=0.025, $k_{m}=2,K=4$ using the MUSE trial data

SLEDAI		PGA		BILAG		Taper							
$\delta_{1}$	$\sigma_{1}^{2}$	$\delta_{2}$	$\sigma_{2}^{2}$	$\left(\pi_{T3},\pi_{C3}\right)$	$\delta_{3}^{*}$	$\left(\pi_{T4},\pi_{C4}\right)$	$\delta_{4}^{*}$	$n_{co}$	$n_{mult}$	$SS_{1}$	$SS_{2}$	$SS_{3}$	$SS_{4}$
0.88	18	0.38	0.35	(0.97,0.95)	0.24	(0.54,0.38)	0.40	403	46	365	39	273	99
0.88	19	0.38	0.35	(0.97,0.95)	0.24	(0.54,0.38)	0.40	419	46	386	39	273	99
0.88	20	0.38	0.35	(0.97,0.95)	0.24	(0.54,0.38)	0.40	435	46	406	39	273	99
0.88	18	0.38	0.45	(0.97,0.95)	0.24	(0.54,0.38)	0.40	403	55	365	49	273	99
0.88	18	0.38	0.55	(0.97,0.95)	0.24	(0.54,0.38)	0.40	403	63	365	60	273	99
0.88	18	0.38	0.65	(0.97,0.95)	0.24	(0.54,0.38)	0.40	403	70	365	71	273	99

*Note*: $SS_{1},SS_{2},SS_{3},SS_{4}$ are sample sizes required per group for the individual endpoints for a power of at least 1−β=0.80.

We assume that a future trial in SLE is to be conducted using the composite responder endpoint, allowing for uncertainty in σ. The estimated variance for the risk difference from the trial dataset is $\sigma_{\delta }^{2}=0.048$ with correlation parameters $\rho_{12}=0.448,\rho_{13}=0.521,\rho_{14}=0.003,\rho_{23}=0.448,\rho_{24}=-0.031,\rho_{34}=0.066$. For a risk difference of 0.14, the required sample size per group is 50, compared to 135 for 88% power in the standard binary method. If the method were to be employed for increased power, rather than a decrease in required sample size, the estimated power of the latent variable method is over 99.99% for sample sizes giving 88% power at the 0.05 one‐sided alpha level in the binary method. The empirical power is shown for the latent variable method in 1000 simulated datasets, which is approximately 88% for each sample size, as required. Note that the sample size for composite endpoints are highly dependent on the responder threshold chosen, which will be predefined by clinicians.

## 5 EMPIRICAL PERFORMANCE OF SAMPLE SIZES

The behavior of the sample sizes obtained for each of the endpoints can be shown empirically. Assuming the four dimensional SLE endpoint, we calculate the empirical power by simulating 100 000 datasets from the multivariate normal distribution and applying the corresponding tests for both the observed and latent continuous outcomes. The key concern for the co‐primary endpoints is that the method gives the appropriate power whereas for multiple primary endpoints we must ensure the family‐wise error rate is controlled.

**FIGURE 3:**
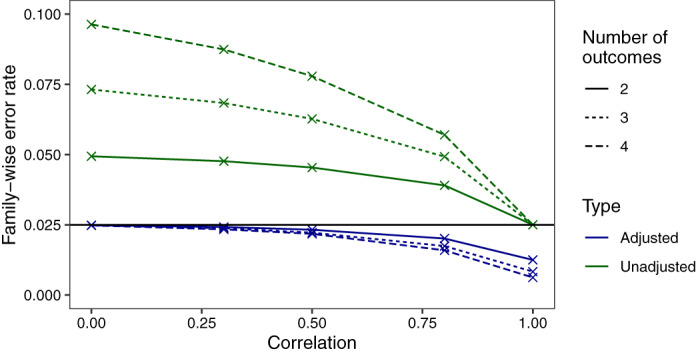
Family‐wise error rate (FWER) of the multiple primary endpoints shown both unadjusted and adjusted using the Bonferroni correction. FWERs are shown for K=(2,3,4) outcomes and correlations are constrained to be equal between all outcomes

The sample sizes required for each of the three endpoints and the corresponding empirical power is shown for effect sizes observed in the MUSE trial with low, medium, and high correlation assumed between endpoints (Table 2). The empirical power derived is approximately equal to the desired power of 80% for all endpoints. As is well recognized in the multiple testing literature, the type I error rate must be controlled when multiple primary endpoints are tested using the union‐intersection test. The degree to which the type I error rate is inflated depends on the number of outcomes and the correlation between outcomes, where lower correlation between outcomes and larger number of outcomes result in larger inflations (Figure 3). The performance of the Bonferroni correction in this setting is shown, where it is conservative in the case of high correlation between endpoints. As the maximum correlation between outcomes in the MUSE trial endpoint used in the numerical example is 0.5, we expect the sample sizes shown for this application to be a good estimate. If very large positive correlations between the endpoints are expected the required sample size from this approach may be overestimated. The code to obtain these empirical results is provided in the ‘Software’ section.

**TABLE 2:** Sample sizes and empirical power (%) for $n=n_{C}=n_{T}$ for the co‐primary, multiple primary, and composite endpoints for overall power 1−β≈0.80, α=0.025, $k_{m}=2,K=4$ with observed and latent effect sizes $\delta_{1},\delta_{2},\delta_{3}^{*},\delta_{4}^{*}$ and correlation ρ equal to 0.3, 0.5, 0.8 where correlations are assumed to be equal between all endpoints

$\delta_{1}$	$\delta_{2}$	$\delta_{3}^{*}$	$\delta_{4}^{*}$	ρ	Co‐primary	Multiple primary	Composite
0.12	0.12	0.12	0.12	0.0	1766 (80.1)	591 (80.0)	1031 (80.1)
				0.3	1692 (80.0)	744 (80.1)	883 (80.0)
				0.5	1617 (80.0)	867 (80.1)	687 (80.0)
				0.8	1439 (80.0)	1117 (80.0)	589 (79.9)
0.35	0.35	0.15	0.15	0.0	917 (79.9)	105 (80.1)	201 (79.9)
				0.3	894 (80.0)	122 (79.9)	156 (80.0)
				0.5	870 (80.0)	134 (80.0)	115 (80.1)
				0.8	815 (80.1)	153 (80.0)	92 (80.0)
0.12	0.35	0.55	0.10	0.0	1772 (80.2)	61 (80.3)	81 (80.0)
				0.3	1736 (80.2)	67 (80.5)	72 (80.1)
				0.5	1700 (80.0)	70 (79.9)	67 (80.2)
				0.8	1625 (80.2)	74 (80.6)	58 (80.1)

## 6 DISCUSSION

The work in this article demonstrated the various ways in which a latent variable framework may be employed for mixed continuous, ordinal, and binary outcomes. We illustrated sample size determination in the case of mixed continuous, ordinal, and binary co‐primary outcomes. We extended this to allow for sample size determination in the case of mixed multiple primary endpoints and proposed a technique to estimate the sample size when using a latent variable model for the underlying structure of a mixed composite endpoint. For co‐primary and multiple primary endpoints the resulting hypothesis is based on an intersection or union of the hypotheses for the individual outcomes and so is multivariate in nature. However, for composite responder endpoints the hypothesis of interest is stated in relation to the overall responder endpoint and so is univariate. Sample size estimation in this case can make use of the standard power and sample size functions but requires the distribution of the test statistic under the alternative hypothesis which we approximate using latent‐level means and a Taylor series expansion.

We applied the methods to a numerical example based on a phase IIb study. For the correlation structure observed in the MUSE trial, the sample size required for the co‐primary endpoint was greater than that required for the individual endpoint with the lowest effect size. Alternatively, the sample size required for the multiple primary endpoint changes based on the variance assumed for the outcome with the largest treatment effect, however is similar to that required by the individual endpoint. The sample size required for the composite endpoint was between that required for the individual outcome with the largest and second largest effect size. Given that in the composite case we are concerned with the overall binary response endpoint, we compared the sample sizes required for the endpoint using the latent variable model with the standard binary method which we showed offered a large gain in efficiency. Results of the simulated scenarios agree with previous findings that the inclusion of the ordinal component with five levels is only responsible for a very small proportion of the precision gains. Given that the inclusion of the ordinal component substantially increases complexity and computational demand, it may be sufficient to combine any ordinal components with the binary outcome if necessary. Detailed simulation results for the composite endpoint are shown in the Supplementary Material.

One practical consideration when calculating the sample size for a trial using the latent variable model is the need to specify a large number of parameters, even in the case of only a few outcomes. Estimates for the parameters could be obtained by fitting the model to pilot data however this is potentially challenging and restrictive for a number of reasons. First, it requires that a pilot or earlier phase trial must have already taken place. Furthermore, the pilot data could be fundamentally different to the future trial and observed effects may be imprecise. Therefore, placing too much emphasis on the existing data may lead to problems in the main trial. In theory, it is possible to specify the required covariance parameters without data however this would be difficult in practice. Additionally, in the case of composite endpoints, we cannot define the variance in terms of the model parameters only, as the treatment effect is defined for the one‐dimensional composite and so is a function of the parameters. This means that the full covariance matrix of the estimated parameters is required for the Taylor series derivation. An alternative when there is no data available is to apply the method using the sample size required to achieve 80% power for the binary method and avail of the large increase in power. Alternatively, we can directly specify $\sigma_{\delta }$ based on expert elicitation, as is sometimes the case in practice for standard one‐dimensional endpoints. Allowing for uncertainty in the quantities and choosing conservative values should provide an appropriate sample size estimate.

It is possible to extend this approach to use adaptive sample size re‐estimation, or an internal pilot to allow for reductions in the required sample size in the trial as we collect more information about the treatment effect variability.

## SOFTWARE

The code to obtain the results in this article is available at https://github.com/martinamcm/mcmenamin_2021_multsamp. A Shiny application for implementing the method is available at https://martinamcm.shinyapps.io/multsampsize/. Documentation and example data are available at https://github.com/martinamcm/MultSampSize.

## Supporting information

Data S1: Supplementary Material
