# Supplementary material for: Sample size estimation using a latent variable model for mixed outcome co‐primary, multiple primary and composite endpoints
Source: Stat Med. 2022 Feb 23;41(13):2303–16. doi: 10.1002/sim.9356 (PMC7612654; doi:10.1002/sim.9356)
Supplement: Supplementary file 1 — Data S1: Supplementary Material [file SIM-41-2303-s001.pdf]

Sample Size Estimation using a Latent Variable Model for Mixed Outcome Co-Primary, Multiple Primary and Composite Endpoints: Supplementary Material

1. Composite endpoints

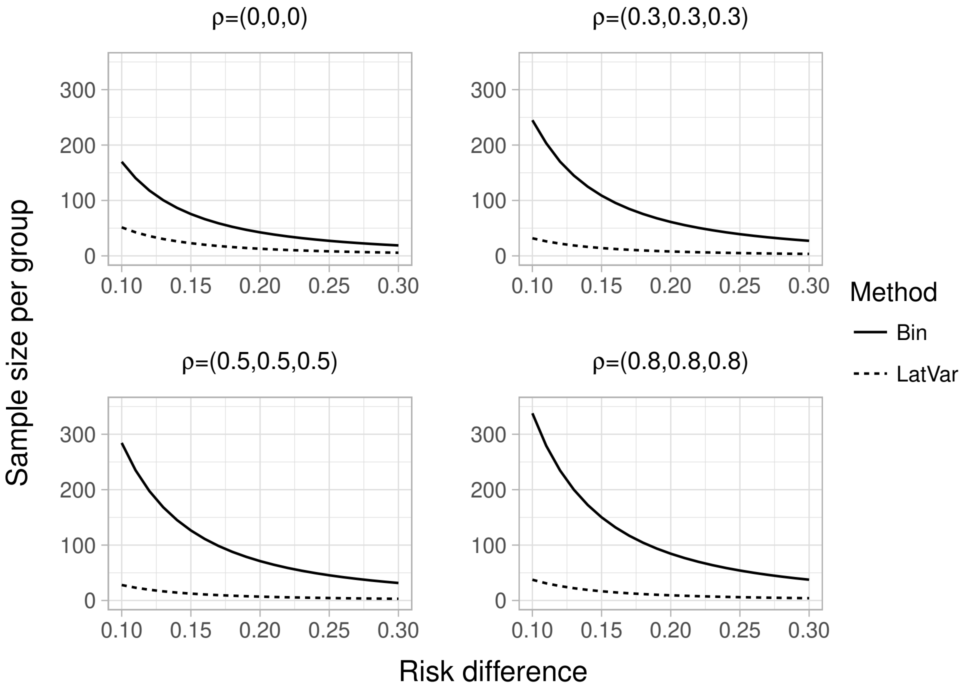

Fig. 1. Estimated sample size per group for different values of the risk difference using the latent variable and standard binary methods when the composite endpoint is formed from one continuous, one ordinal and one binary outcome, where all components drive response and correlations between the outcomes are between 0 and 0.8, where $\rho = (\rho_{12}, \rho_{13}, \rho_{23})$

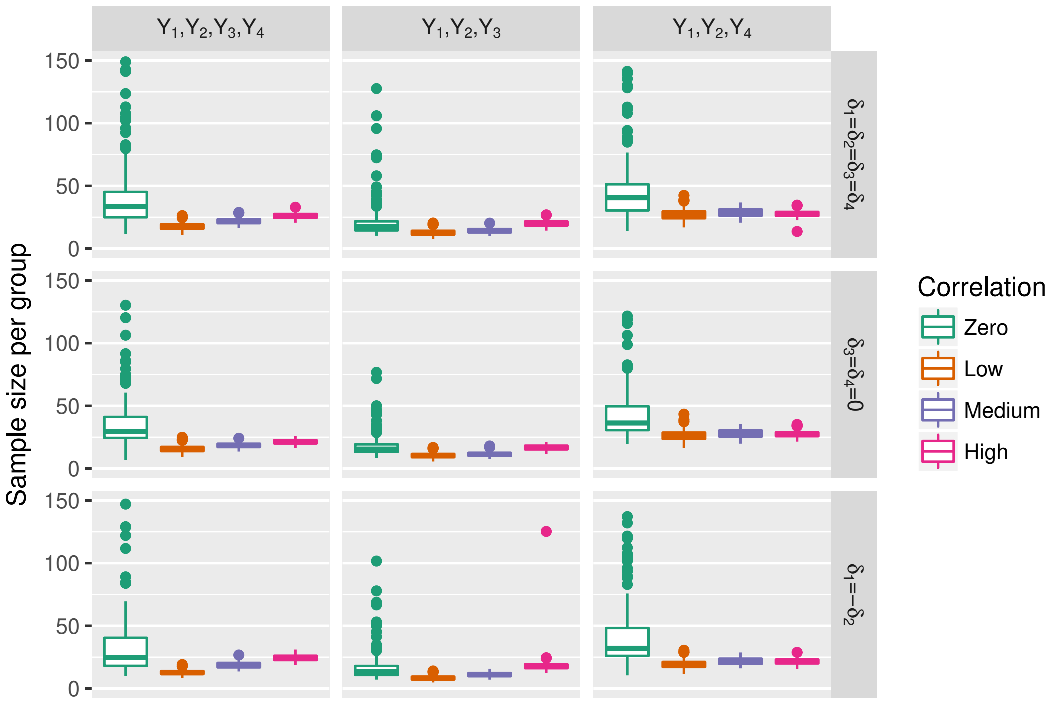

Fig. 2. Boxplots of the estimated sample size per group from 1000 simulated datasets using the latent variable method for composites containing two continuous, one ordinal and one binary component and correlation between endpoints is zero, low=0.3, medium=0.5 or high=0.8. These are shown when response is driven by (Y_1, Y_2, Y_3, Y_4), (Y_1, Y_2, Y_3) or (Y_1, Y_2, Y_4) and the treatment effect structure in the components is $\delta_1 = \delta_2 = \delta_3 = \delta_4$, $\delta_3 = \delta_4 = 0$ or $\delta_1 = -\delta_2$

Table 1. Median sample sizes $n = n_C = n_T$ for overall power $1-\beta \approx 80\%$, $\alpha = 0.05$, $k_m = 1, K = 3$, $\delta = \mu_T - \mu_C$: overall risk difference on the composite, $\delta^\dagger$: treatment effect structure in the components, for a combination of correlations 0, L=0.3, M=0.5, H=0.8 using the latent variable model when the composite is made up of one continuous, one ordinal and one binary outcome

Response	$\delta^\dagger$	δ	Correlation $(\rho_{12}, \rho_{13}, \rho_{23})$						
			000	00H	LLL	LLH	MMM	MMH	HHH
Y_1, Y_2, Y_3	$\delta_1 = \delta_2 = \delta_3$	0.05	206	339	128	119	112	108	145
		0.10	52	85	32	30	28	27	38
		0.15	23	38	15	14	13	12	17
	$\delta_3 = 0$	0.05	201	334	123	117	105	112	159
		0.10	51	84	31	30	27	28	40
		0.15	23	38	14	13	12	13	18
	$\delta_2 = \delta_3 = 0$	0.05	197	329	119	114	101	106	121
		0.10	49	81	29	29	27	21	30
		0.15	21	36	14	13	12	12	15
Y_1, Y_3	$\delta_1 = \delta_2 = \delta_3$	0.05	474	494	289	200	267	205	240
		0.10	119	124	73	50	67	52	60
		0.15	53	55	33	23	30	23	27
	$\delta_3 = 0$	0.05	468	499	286	195	264	203	248
		0.10	117	125	72	49	66	51	62
		0.15	52	56	32	22	30	23	28
	$\delta_2 = \delta_3 = 0$	0.05	470	501	287	196	264	204	249
		0.10	119	123	73	49	67	51	63
		0.15	53	55	32	23	31	24	28
Y_3	$\delta_1 = \delta_2 = \delta_3$	0.05	1493	1472	1250	1113	948	793	609
		0.10	374	368	313	279	237	199	153
		0.15	166	164	139	124	106	89	68
	$\delta_3 = 0$	0.05	1502	1468	1256	1113	960	806	622
		0.10	376	367	315	279	240	202	156
		0.15	176	164	140	124	107	90	70
	$\delta_2 = \delta_3 = 0$	0.05	1504	1465	1259	1115	963	807	624
		0.10	376	370	316	280	241	203	156
		0.15	174	164	139	126	106	90	70

Table 2. Median sample sizes $n = n_C = n_T$ for overall power $1 - \beta \approx 80\%$, $\alpha = 0.05$, $k_m = 1, K = 3$, $\delta = \mu_T - \mu_C$: overall risk difference on the composite, $\delta^\dagger$: treatment effect structure in the components, for a combination of correlations ranging from 0, L=0.3, M=0.5, H=0.8 using the standard binary method when the composite is made up of one continuous, one ordinal and one binary outcome

Response	$\delta^\dagger$	δ	Correlation $(\rho_{12}, \rho_{13}, \rho_{23})$						
			000	00H	LLL	LLH	MMM	MMH	HHH
Y_1, Y_2, Y_3	$\delta_1 = \delta_2 = \delta_3$	0.05	680	965	980	1141	1138	1214	1352
		0.10	170	242	245	286	285	304	338
		0.15	76	108	109	127	127	135	151
	$\delta_3 = 0$	0.05	628	939	928	1098	1102	1183	1332
		0.10	157	235	232	275	276	296	333
		0.15	70	105	104	122	123	132	148
	$\delta_2 = \delta_3 = 0$	0.05	609	920	914	1086	1097	1171	1310
		0.10	147	231	228	270	271	290	328
		0.15	68	101	101	119	121	130	146
Y_1, Y_3	$\delta_1 = \delta_2 = \delta_3$	0.05	1127	1136	1255	1261	1334	1320	1425
		0.10	282	284	314	316	334	330	357
		0.15	126	127	140	141	149	147	159
	$\delta_3 = 0$	0.05	1078	1072	1216	1218	1298	1296	1403
		0.10	270	268	304	305	325	324	351
		0.15	120	120	136	136	145	144	156
	$\delta_2 = \delta_3 = 0$	0.05	1066	1063	1202	1209	1296	1297	1403
		0.10	263	259	300	299	319	324	351
		0.15	121	119	133	131	143	143	156
Y_3	$\delta_1 = \delta_2 = \delta_3$	0.05	1547	1548	1550	1548	1550	1550	1549
		0.10	387	387	388	387	388	388	388
		0.15	172	172	173	172	173	173	173
	$\delta_3 = 0$	0.05	1544	1545	1549	1548	1549	1548	1545
		0.10	386	387	388	387	388	387	387
		0.15	172	172	173	172	173	172	172
	$\delta_2 = \delta_3 = 0$	0.05	1544	1546	1549	1546	1551	1549	1545
		0.10	386	389	387	385	388	388	387
		0.15	173	172	173	173	172	172	172

Table 3. Median sample sizes per group $n = n_C = n_T$ for overall power $1 - \beta \approx 80\%$, $\alpha = 0.05$, $k_m = 2, K = 4$, $\delta = \mu_T - \mu_C$: overall risk difference on the composite , $\delta^\dagger$: treatment effect structure in the components, for a combination of correlations 0, L=0.3, M=0.5, H=0.8 using the latent variable model when the composite is comprised of two continuous, one ordinal and one binary outcome

Response	$\delta^\dagger$	δ	Correlation $(\rho_{12}, \rho_{13}, \rho_{14}, \rho_{23}, \rho_{24}, \rho_{34})$			
			000000	LLLLLL	MMMMMM	HHHHHH
Y_1, Y_2, Y_3, Y_4	$\delta_1 = \delta_2 = \delta_3 = \delta_4$	0.05	70	51	41	81
		0.10	18	13	11	21
		0.15	8	6	5	9
	$\delta_3 = \delta_4 = 0$	0.05	62	41	47	67
		0.10	16	11	12	17
		0.15	7	5	6	8
	$\delta_1 = -\delta_2$	0.05	55	34	45	72
		0.10	14	9	12	18
		0.15	7	4	5	8
Y_1, Y_2, Y_3	$\delta_1 = \delta_2 = \delta_3 = \delta_4$	0.05	139	71	63	105
		0.10	35	18	16	27
		0.15	16	8	7	12
	$\delta_3 = \delta_4 = 0$	0.05	120	63	75	85
		0.10	30	16	19	22
		0.15	13	7	9	10
	$\delta_1 = -\delta_2$	0.05	105	50	76	99
		0.10	27	13	19	25
		0.15	12	6	9	11
Y_1, Y_2, Y_4	$\delta_1 = \delta_2 = \delta_3 = \delta_4$	0.05	166	106	105	112
		0.10	42	27	27	28
		0.15	19	12	12	13
	$\delta_3 = \delta_4 = 0$	0.05	147	105	113	111
		0.10	37	27	29	28
		0.15	17	12	13	13
	$\delta_1 = -\delta_2$	0.05	132	78	88	86
		0.10	33	20	22	22
		0.15	15	9	10	10

Table 4. Median sample sizes per group $n = n_C = n_T$ for overall power $1-\beta \approx 80\%$, $\alpha = 0.05$, $k_m = 2, K = 4$, $\delta = \mu_T - \mu_C$: overall risk difference on the composite, $\delta^{\dagger}$: treatment effect structure in the components, for a combination of correlations 0, L=0.3, M=0.5, H=0.8 using the standard binary method when the composite is comprised of two continuous, one ordinal and one binary outcome

Response	$\delta^{\dagger}$	δ	Correlation $(\rho_{12}, \rho_{13}, \rho_{14}, \rho_{23}, \rho_{24}, \rho_{34})$			
			000000	LLLLLL	MMMMMM	HHHHHH
Y_1, Y_2, Y_3, Y_4	$\delta_1 = \delta_2 = \delta_3 = \delta_4$	0.05	386	739	665	1240
		0.10	97	185	167	310
		0.15	43	83	74	138
	$\delta_3 = \delta_4 = 0$	0.05	324	665	867	1205
		0.10	81	167	217	302
		0.15	36	74	97	134
	$\delta_1 = -\delta_2$	0.05	331	666	858	1169
		0.10	83	167	215	293
		0.15	37	74	96	130
Y_1, Y_2, Y_3	$\delta_1 = \delta_2 = \delta_3 = \delta_4$	0.05	690	956	912	1300
		0.10	173	239	228	325
		0.15	77	107	102	145
	$\delta_3 = \delta_4 = 0$	0.05	650	912	1053	1283
		0.10	163	228	264	321
		0.15	73	102	117	143
	$\delta_1 = -\delta_2$	0.05	605	866	1017	1232
		0.10	152	217	255	308
		0.15	68	97	113	137
Y_1, Y_2, Y_4	$\delta_1 = \delta_2 = \delta_3 = \delta_4$	0.05	690	962	919	1298
		0.10	173	241	230	325
		0.15	77	107	103	145
	$\delta_3 = \delta_4 = 0$	0.05	642	919	1058	1281
		0.10	161	230	265	321
		0.15	72	103	118	143
	$\delta_1 = -\delta_2$	0.05	610	876	1007	1225
		0.10	153	219	252	307
		0.15	68	98	112	137
